# The Role of Liquid-crystalline Structures in the Morphogenesis of Animal Fibers

**DOI:** 10.4103/0974-7753.77516

**Published:** 2010

**Authors:** A John McKinnon, Duane P Harland

**Affiliations:** Vernon Willey Trust Fellow, 80 Mansfield Avenue, Christchurch 8014, NewZealand; 1 Lincoln Research Center, AgResearch Ltd., Private Bag 4749, Christchurch 8140, NewZealand

**Keywords:** Follicle, intermediate filament, macrofibril, mesophase, unit-length filament

## Abstract

The role of liquid-crystalline (mesophase) structures in extra-cellular morphogenesis is widely recognized. This paper summarizes a model for the more unusual case of intra-cellular mesophases. In the nascent mammalian hair cortex, cell differentiation is correlated with different mesophase textures within tactoids that are composed of intermediate filaments (IFs), and which form by a concerted process of unit-length-filament (ULF) polymerization and phase separation. Nematic and double-twist textures arise from differences in mesogen orientation and length in apposed tactoids. The model explains features of mature structures such as the fibril-matrix ratios in different cell types. The rapidity of IF formation suggests that a sudden-transition equilibrium polymerization, involving a high-energy initiating species, obeying the same statistical model as several other biological transitions, may be involved. This leads to an appealing symmetry, with the key factor in both polymerization and mesophase stability being the retention of protein head-group entropy.

Although the role of liquid-crystalline (mesophase) structures in extracellular morphogenesis is widely recognized, instances of such self-organization in intracellular processes are rather more obscure. Transmission electron microscopy (TEM) studies of animal fiber follicle cells [Figure 1] give clear evidence of the formation of a phase-separated developing intermediate filament (IF) material, which appears to have all the characteristics of lyotropic liquid crystal tactoids. Here, we elaborate on previous studies treating the early stages of keratin fibril assembly as a process of mesophase separation,[1] during which IFs align into organized fibrillar templates of what will become macrofibrils. In addition, we couple phase separation with the process of end-to-end ‘annealing’ (polymerization) into IFs of unit-length filament (ULF) structural units, assembled from dimeric and tetrameric keratin protein precursors, adopting a model first proposed by Flory and co-workers in 1978, for such a concerted process.[2] The suddenness and extent of keratin filament formation and variations within the cell types, suggest that polymerization is initiated after the concentrations of the ULF species and the precursors have become quite high.

**Figure 1:**
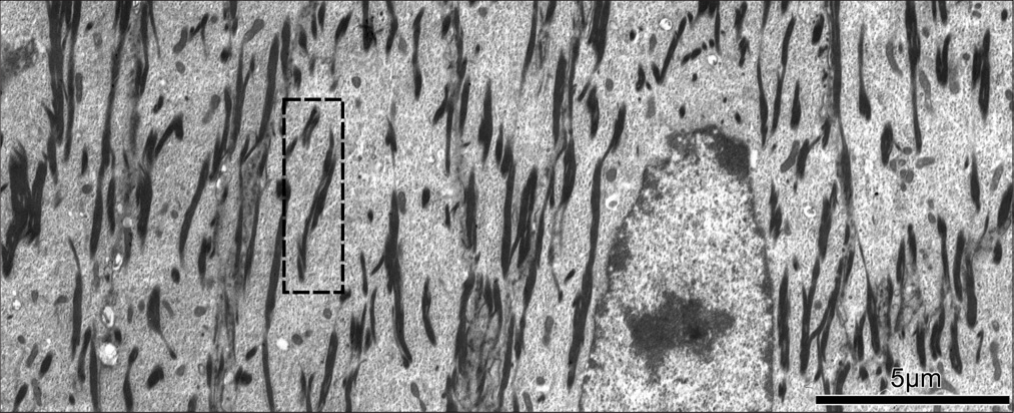
Transmission electron micrograph of the developing wool fiber in longitudinal section (follicle low zone C). Inset box, one example of two macrofibrils coalescing from several smaller tactoids. (Original micrograph courtesy J. L. Woods)

IFs have persistence lengths in the order of several hundred nanometres,[3] comparable with those of such well-known mesogens as DNA and the polysaccharides xanthan and schizophyllan. Given the appropriate solvent interactions, it is to be expected that, as their concentration in the cytoplasm rises, they will undergo phase separation into an oriented, anisotropic phase. This process has been interpreted through the extensive work of Flory and colleagues, based on the lattice theory of phase separation in solutions of rod-like particles.[4] High concentrations of filament precursor species (ULFs and sub-units) in the cytoplasm reduce the length and concentration of the filaments at which phase separation occurs, and produce a more concentrated mesophase, while also increasing the biphasic gap (difference in concentration of the anisotropic and isotropic phases). Wool-water interaction studies reveal that the interaction with the helical filament core is endothermic;[5] the mesophase must then be entropically stabilized, a feature which is of widespread importance in macromolecular mesophases, and we propose that this arises from the conformational and mixing entropy of the protein head groups with water. Such entropic effects contract the biphasic gap. There are thus several competing effects in play in the cytoplasm, which control the conditions under which filaments phase-separate, and thus the length, and length distribution, of the filaments. It is clear from the initial tactoid sizes that separation commences when IFs are very short, only a few ULFs long.

For ULF polymerization, we have adopted a model similar to that demonstrated by Kirmse *et al*.[6] and Portet *et al*.,[7] in their studies on the formation of vimentin IFs, which is essentially an equilibrium polymerization. However, the process of continuous phase transfer results in an inherently non-equilibrium system, and polymerization equilibrium is never reached; it is largely immaterial what the specifics of the polymerization reaction are.

In certain animal fibers, such as merino wool, cortical cell differentiation is quite distinct and may be resolved into the ortho-, meso-, and para-cortex.[8] The respective structural motifs may be related to recognized systems of mesophase organization and behavior, and to the timing of the onset of polymerization. Thus, the ortho-cortex, in which concentric shells of IFs are helically wound about a central axis, is formed from a mesophase double-twist structure, in the form of a blue-phase cylinder, composed of short IF mesogens, phase-separated from a concentrated ULF precursor mixture, in a relatively delayed process. Short mesogens relieve the torsional strains that occur in such a twisted structure. Short mesogens, expelled from a concentrated isotropic phase, also create a concentrated mesophase able to express chiral twisting forces. The resulting macrofibrils, which cannot fuse laterally because of incompatible directions at the periphery, ultimately mature as cylinders and have a high density of filaments, as observed.[8] The meso-cortex is formed from filaments of intermediate length, arising from polymerization in a somewhat less concentrated cytoplasm, which forms a nematic mesophase, derived from tactoids, which are able to coalesce into a continuous, axially disorganized, columnar hexagonal structure, with an intermediate filament / matrix ratio. The para-cortex is formed from still longer, more widely separated filaments, which cannot reorganize to form a continuous phase, so the para-cortex remains predominantly a compression of tactoids, with only partial fusion at their initial apposed zones. Many aspects of such behavior may be summed up as ‘emulsion science in cylindrical co-ordinates’. Thus, the mesophase model is able to rationalize many of the observed features of the cortical structure.

The between-cell differentiations in liquid-crystal structure development, plus the observed temperature sensitivity of IF formation *in vitro*, suggest that filament formation is a delayed sudden-transition event, exhibiting the equivalent of ceiling temperature characteristics. Such a polymerization can be modeled by a variation of standard equilibrium polymerization involving two equilibria,[9] the first relating to the generation of a high-energy activated species which initiates the subsequent reaction. Although we cannot vary the polymerization temperature *in vivo*, we can vary the conjugate thermodynamic variable, namely the negative entropy change on polymerization. Consideration of the thermodynamics of ULF formation, in terms of the Van’t Hoff equation, suggests that this is the most important parameter. We therefore propose that the high energy initiating species is a ULF, which has undergone an axial displacement, initiating the succeeding addition of overlapped ULFs [Figure 2]. The result of this axial displacement is a reduction of the entropy change on polymerization, brought about by conservation of configurational and mixing entropies in the filament, which also stabilize the mesophase. Such a model, therefore, has an appealing symmetry, in that it accounts for the mesophase stability as well as the sudden onset of polymerization. One might suggest that the activated state is created by the expression of a particular keratin protein with specific head group characteristics, in which entropic interactions between the dimer head groups push the ULF into its axially displaced form.

**Figure 2:**
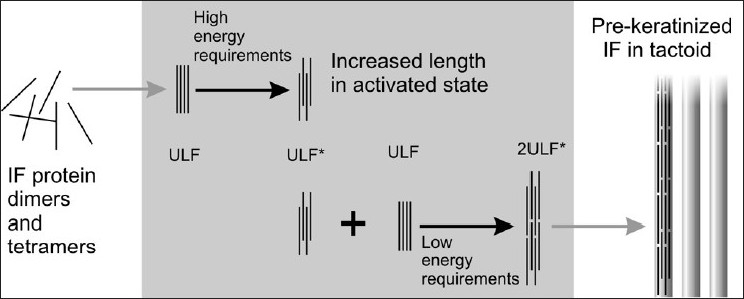
Stylized illustration of IF formation, showing our proposed mechanism of the generation of multi-ULF precursors in the central box. Activated ULFs are indicated by an asterisk. A 32-chain model for the trichokeratin ULF is assumed

This sudden transition model can be equally described in a statistical thermodynamic treatment,[10] which turns out to have the same formalism as the statistical models describing such well-known biological transitions as helix-coil or α→β conversions. It thus appears to have features that give it plausibility in a biological context.
